# Evaluation of antimycobacterial, leishmanicidal and antibacterial activity of three medicinal orchids of Arunachal Pradesh, India

**DOI:** 10.1186/s12906-017-1884-z

**Published:** 2017-08-01

**Authors:** Manisha Bhatnagar, Nandan Sarkar, Nigam Gandharv, Ona Apang, Sarman Singh, Sabari Ghosal

**Affiliations:** 1 Center for Plant and Environmental Biotechnology, Amity Institute of Biotechnology, Amity University, Noida, 201303 India; 2 Department of Laboratory Medicine, All India Institute of Medical Sciences, New Delhi, 110029 India; 3 Department of Environment & Forest, State Forest Research Institute, Van Vihar, Itanagar, Arunachal Pradesh 791111 India

**Keywords:** *Rhynchostylis retusa*, *Tropidia curculioides*, *Satyrium nepalense*, Antimycobacterial, Antileishmanial, Antibacterial

## Abstract

**Background:**

The ethnic population of Arunachal Pradesh uses a number of orchids as such, or in decoction for various ailments. Three untapped orchids namely, *Rhynchostylis retusa, Tropidia curculioides* and *Satyrium nepalense,* traditionally used in tuberculosis, asthma and cold stage of malaria in folk medicine, were selected for the present study.

**Methods:**

Dried material of each plant was divided into three parts. Solvent extraction and fractionation afforded altogether 30 extracts and fractions, which were evaluated against *Mycobacterium tuberculosis* (H37Rv and MDR strain) for antimycobacterial activity; promastigotes and amastigotes of *Leishmania donovani* for leishmanicidal activity and two gram positive and three gram negative clinical isolates for antibacterial activity.

**Results:**

The most significant antimycobacterial activity was observed with *n*-hexane fraction of the flower of *Satyrium nepalense* with MIC of 15.7 μg/mL. The most promising leishmanicidal activity was observed with diethyl ether fraction of the roots of *Rhynchostylis retusa* with IC_50_ values of 56.04 and 18.4 μg/mL against promastigotes and intracellular amastigotes respectively. Evaluation of antibacterial activity identified *S. nepalense* flower *n*-hexane and *R. retusa* roots diethyl ether as potential fractions with MIC values of ≤100 μg/mL against selected clinical isolates.

**Conclusions:**

This is the first report of the plants possessing antimycobacterial and leishmanicidal activity. The investigation resulted in identification of *S. nepalense* as the most promising plant, which possessed all three activities in significant proportion. This laboratory outcome could be translated to marketable pharmaceutical products and also to produce maximum benefits to the local of nearby area.

**Graphical abstract:**

Antimycobacterial and leishmanicidal activity of medicinal orchids
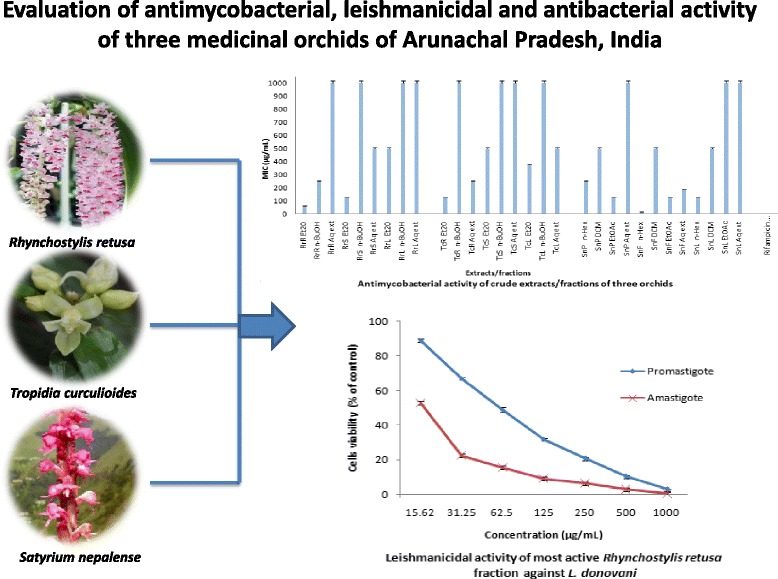

## Background

India harbors a rich repository of untapped medicinal plants, with plenty of associated knowledge that needs to be appropriately utilized. The vast degree of diversity present in the country is directly related to the highly divergent ecosystem and altitudinal variations [1]. Proper scientific investigation of the unexplored natural resources and subsequent commercialization could bring benefits to the stakeholders and also could play a central role in the drug development programs.

Arunachal Pradesh (AR) is a natural habitat of 5000 flowering species including 550 species of orchid plants. Further, it is interesting to note that nearly 300 species of the total orchids are rare whereas, only 37 species are of medicinal importance [2]. Medicinal orchids prefer tropical climate though, a few of them grow under extreme climatic conditions also. Considering serenity of the habitat of AR and traditional use of orchids by the locals, we selected three relatively unexplored plants including *Rhynchostylis retusa* (Rr)*, Tropidia curculioides* (Tc), and *Satyrium nepalense* (Sn) for the present investigation*. R. retusa* of the genus Rhynchostylis is widely distributed all over North-eastern Himalayas and specially linked with the people of Assam, commonly called ‘Kopu Phool’ in Assamese language. It is a symbol of fertility and merriment [3]. The roots of *R. retusa* are used in folk medicine for rheumatism, asthma, tuberculosis, nervous twitchings, cramps, infantile epilepsy, vertigo, palpitation, kidney stone, and menstrual disorders [4]. *T. curculioides* is an endangered plant commonly found in Sikkim and Arunachal Pradesh. The decoction of roots of *T. curculioides* is used for cold stage of malaria and diarrhoea [5]. *S. nepalense* (Hathjadi), a long terrestrial herb (25-60 cm) is commonly found at 2400–5000 m height. The tubers of *S. nepalense* are consumed by the native people as food, tonic and aphrodisiac [6]. The plant is reported to have beneficial effects in the symptom of diarrhoea, malaria and dysentery [7]. Qualitative phytochemical analysis and antimicrobial screening had been conducted with the plant extracts of *R. retusa* [8, 9]. However, any literature related to phytochemical investigation and biological activities of *T. curculioides* was not available. The presence of quercetin, had been reported from the extracts of *S. nepalense* [10]*.* The literature search report inspired us to investigate the medicinal property of the plants.

The folk medicines of this region are used quite extensively for a number of common ailments and communicable diseases [11]. Isolated living environment and poor access to healthcare is a threat for survival of the ethnic population all over India. Especially, the management of highly prevalent communicable diseases for e.g., tuberculosis (TB), visceral leishmaniasis (VL) and malaria is a great concern. TB in humans is caused by *Mycobacterium tuberculosis,* a facultative intracellular microbe belonging to *M. tuberculosis* complex. Further, the alarming rise of multi-drug-resistant (MDR), extensively drug-resistant (XDR) and totally drug resistant (TDR) strains of *M. tuberculosis,* emphasizes the need of new leads based on traditional knowledge [12]. Visceral leishmaniasis, also known as kala azar is a fatal vector-borne illness caused by *Leishmania donovani.* It is the second most dreaded parasitic disease after malaria, causing considerable morbidity and mortality. Interestingly, a number of plant extracts and plant derived compounds are reported to possess significant leishmanicidal activity [13] however, very few have reached to the stage of clinical trials. Also, the dramatic increase of new and emerging MDR bacterial strains emphasizes the need of fresh investigation with those natural resources, which has not been examined yet.

## Methods

### Plant materials

The plants were collected from the foothills of Tipi & Khellong, and Doimara and Sange district of Arunachal Pradesh. The details of geographical location, altitude and place of collection have been mentioned in Table 1. The plants were authenticated by Dr. Ona Apang, State Forest Research Institute, Itanagar, Arunachal Pradesh. The voucher specimens of *R. retusa* (AUUP/AIB/2014/01), *T. curculoidies* (AUUP/AIB/2014/02) and *S. nepalense* (AUUP/AIB/2015/03) were preserved in the herbarium of Amity Institute of Biotechnology, Amity University, Noida.

**Table 1:** Orchid species collected from Arunachal pradesh (AR); their geographical location, medicinal use and conservation status

S.No.	Name of species and voucher no.	Place of collection	Geographical location	Altitude (m)	Medicinal use	Conservation status
1	*Rhyncostylis retusa* AUUP/AIB/2014/01	Doimara (AR)	N 26^0^58′02.0″ & E 092^0^25′10.3″	357	Rheumatism, asthma, tuberculosis, nervous twitchings, cramps, infantile epilepsy	Vulnerable
2	*Tropidia curculioides* AUUP/AIB/2014/02	Tippi (AR)	N 27^0^0′14.9″ & E 092^0^36′44.2″	339	Malaria and diarrhoea	Rare
3	*Satyrium nepalense* AUUP/AIB/2014/03	Sange (AR)	N 27^0^27′30.1″ & E 092^0^06′17.4″	3072	Malaria, dysentery and aphrodisiac	Endemic

### Chemicals and reagents

The solvents were purchased from Merck, India. Middlebrook 7H9 (Becton-Dickinson) supplemented with 0.1% casitone, 0.2% glycerol and 10% OADC (oleic acid, albumin, dextrose and catalase), Resazurin dye [Resazurin sodium salt (Sigma®, USA)], MTT [3-(4,5-dimethylthiazol-2-yl)-2,5 diphenyltetrazolium bromide] Sigma-Aldrich, 96-well plate with lid (Becton Dickinson), Acrodisc Syringe filter (Pall Life Sciences) with 0.2 μm filters, >95% pure rifampicin (RIF), amphotericin B and miltefosine standard (≥98%) (Sigma-Aldrich) were used to conduct the following biological experiments. Pre-coated silica gel 60 F_254_ plates were used for analytical TLC.

### Extraction and fractionation

The dried plant material of *R. retusa* and *T. curculioides* were divided into root, stem and leaves while, *S. nepalense* was divided into pseudobulb, stem and leaves, and flowers. Dry weight of each part was noted and was extracted with methanol: water (9:1, 1 L× 24 h) for three consecutive days followed by extraction of the residue with H_2_O (0.5 L) at room temperature. The concentrated MeOH extracts of *R. retusa* and *T. curculioides* were suspended in water and was fractionated into diethyl ether (Et_2_O), *n*-BuOH and residual aqueous fractions successively. As, the plant material of *S. nepalense* was available in larger amount (4.23 Kg) compared to the other plants, more elaborate fractionation was carried out with the MeOH extract to obtain four fractions including *n*-hexane (*n*-Hex), dicholoromethane (DCM) and ethyl acetate (EtOAc) and residual aqueous fractions.. The organic solvents were removed under reduced pressure, below 40 °C in a rotary evaporator. Subsequently, a portion of aqueous extracts were lyophilized and refrigerated at 4 °C. Thin Layer Chromatography (TLC) profile of the active fractions was evaluated at varying polarity of ethyl acetate and *n*-Hex on pre-coated TLC plates.

### Phytochemical screening of extracts

Phytochemical analysis for the presence of alkaloids, flavonoids, steroids, reducing sugars, cardiac glycosides, terpenoids, anthraquinones, tannins, phlobatanins and saponins were conducted with each fraction by using standard protocol [14].

### Colorimetric redox indictor assay (CRI assay)

The pan sensitive strain of *M. tuberculosis* H37Rv TMC-102 (sensitive to streptomycin, isoniazide, rifampicin, ethambutol and pyrazinamide) and another MDR clinical isolate (Tb-14,348/16), resistant to rifampicin and isoniazid, was obtained as gift from Dr. V.M. Katoch, National JALMA Institute of Leprosy and other Mycobacterial Diseases, Agra, India. Briefly, the bacterium at log phase of growth (approximately 12 days), was transferred to a sterile vial containing glass beads and 8 mL of sterile 0.85% saline solution. The bacterial suspension was disaggregated by agitation and was allowed to stand for 15 min at room temperature. Turbidity of the suspension was compared with 1 McFarland tube standard and was adjusted with 7H9 broth to obtain a bacterial concentration of 3 × 10^8^ CFU/mL. The working solution at 1:20 dilution of the suspension, in Middlebrook 7H9 broth was evaluated by Colorimetric Redox Indicator Assay (CRI assay) [15]. The extracts were dissolved in DMSO and were diluted appropriately to obtain final sample concentrations in the range of 100–500 μg/mL. The bacterial suspension at a concentration of 1.5 × 10^8^cells/mL was added to each well where, media along with bacterial suspension was considered as growth control and 0.5% DMSO with media and bacterial suspension were used as DMSO control. Besides, the well known antimycobacterial agent rifampicin was used as positive control. Sterile water was added to all perimeter wells to avoid evaporation. The microplates were incubated for 5–7 days at 37 °C in an incubator followed by addition of 25 μL of resazurin (0.02% *w*/*v*) dye to each well. Then, the plates were re-incubated at 37 °C for 24 h for color development. The minimum inhibitory concentration (MIC) was defined as the lowest drug/extract concentration that prevented color change of resazurin reagent from blue to pink. Blue color is interpreted as no mycobacterial growth and pink color as growth occurrence [16]. All the experiments were carried out in duplicate with three independent experiments.

### Leishmanicidal assay

Pan sensitive strain of *L. donovani* (DD8) was obtained from Department of Laboratory Medicine (AIIMS), New Delhi, India. The culture was routinely maintained at 24 °C in M-199 (GIBCO®, USA) medium supplemented with penicillin (100 U/mL), streptomycin (100 μg/mL) (Invitrogen, USA) and 10% heat inactivated fetal calf serum (FCS; GIBCO®, USA).

Promastigotes at logarithmic phase were seeded (1 × 10^6^ cells/mL) in 96-well microtiter plate in the presence of different concentrations of samples and incubated at 24 °C for 48 h. Thereafter, 100 μl of MTT (5 mg/mL) solution was added to each plate and incubated for 4 h at 24 °C. Finally, 100 μl of DMSO was added in each well to dissolve the formazan produced, followed by 18 h of incubation. The absorbance was measured at 570 nm and DMSO (0.5%) was considered as control while, amphotericin B and miltefosine was used as reference. Each assay was performed in duplicate with three independent experiments [17].

To produce intracellular amastigotes, J774G8 (5 × 10^5^cells/mL) macrophage cells were plated onto 13-mm coverslips in 24-well plates for 1 h at 37 °C in a CO_2_ incubator. Non adherent cells were removed and the cells were further incubated overnight. Adherent cells were infected with *L. donovani* promastigotes at a parasite: macrophage ratio of 10: 1 and further incubated for 1 h. Unbound promastigotes were removed by extensive washing with PBS (pH 7.2). The infected macrophages were incubated with various dilutions of samples and the mean percentage of viable amastigotes was calculated relative to control and the results were expressed as concentration inhibiting the parasitic growth. The leishmanicidal effect of each sample was expressed in IC_50_ values.

### Cell cytotoxicity assay

The cell cytotoxicity was assessed against J774G8 murine macrophage cells (1 × 10^6^ cells/mL) with different concentrations of active fractions in the range of 15.62–250 μg/mL. The cell viability was determined by MTT assay [18] as well. The results were expressed as percentage reductions in cell viability, compared to untreated control wells. The cytotoxic concentration required to kill 50% of the cells (CC_50_) was calculated.

### Antibacterial assay

Five MDR bacterial clinical isolates of *Staphylococcus aureus* (2413), *Enterococcus* sp. (2449), *Serratia sp*. (2442), *Acinetobacter* sp. (2457) and *Escherichia coli* (2461) were obtained with respective antibiotic resistance profiles from Dr. Kumardeep Dutta Choudhary, Department of Medical Oncology, Rajiv Gandhi Cancer Research Institute, Delhi, India. All bacterial strains were revived in nutrient broth to conduct antibacterial screening [19]. Briefly, nutrient agar plates were inoculated with 0.1 mL of each organism (1 × 10^8^ CFU/mL) and were treated with 50 μL of samples. The plates were incubated at 37 °C for 24 h. The antimicrobial activity was expressed as the mean diameter of inhibition zones (mm) with standard deviation produced by the tested fractions. Tetracycline and gentamycin were used as positive controls for disc diffusion assay. Minimum inhibitory concentrations (MIC) values were determined for the most potent extract by tube dilution method [20]. A series of two fold dilutions of each extract ranging from 1 mg/mL to 0.1 mg/mL were done in Muller Hilton broth and were inoculated with 0.1 mL of suspension of the test organism. The tubes were incubated at 37 °C for 24 h and checked for turbidity. Minimum inhibitory concentration was determined as highest dilution of the extract that showed no visible growth.

## Result and discussion

Solvent extraction and fractionation of the plants afforded 30 components, dry weight of which is demonstrated in Table 2. The results of qualitative analysis for different classes of phytochemicals viz., alkaloids, flavonoids, steroids, reducing sugar, cardiac glycosides, terpenoids, anthraquinones, tannins, phlobatanins and saponins had been presented in Table 3. The phytochemical analysis data provides an overview of the chemical classes and their relative proportion in a fraction. Further, this knowledge could serve as a background to select the isolation strategy of the active ingredients.

**Table 2:** Details of extraction and fractionation of medicinal orchids obtained from AR

S.No.	Name	Part used for fractionation	Fraction/Extract	Yield (g)
1	*Rhynchostylis retusa* (Rr)- 2.34 kg	Roots (1.178 kg)	diethyl ether fraction (RrR Et_2_O)	18.52
			*n-*butanol fraction (RrR *n*-BuOH)	12.77
			aqueous extract (RrR Aq ext)	5.00
		Stem (0.354 kg)	diethyl ether fraction (RrS Et_2_O)	8.44
			*n*-butanol fraction (RrS *n*-BuOH)	4.82
			aqueous extract (RrS Aq ext)	11.80
		Leaves (0.726 kg)	diethyl ether fraction (RrL Et_2_O)	6.28
			*n*-butanol fraction (RrL *n*-BuOH)	10.92
			aqueous extract (RrL Aq ext)	7.01
2	*Tropidia curculioides* (Tc) - 1.622 Kg	Roots (0.922 kg)	diethyl ether fraction (TcR Et_2_O)	5.18
			*n*-butanol fraction (TcR *n*-BuOH)	16.46
			aqueous extract (TcR Aq ext)	38.99
		Stem (0.302 kg)	diethyl ether fraction (TcS Et_2_O)	2.13
			*n*-butanol fraction (TcS *n*-BuOH)	15.62
			aqueous extract (TcS Aq ext)	4.19
		Leaves (0.310 kg)	diethyl ether fraction (TcL Et_2_O)	16.00
			*n*-butanol fraction (TcL *n*-BuOH)	11.96
			aqueous extract (TcL Aq ext)	1.65
3	*Satyrium nepalense* (Sn)- 4.233 kg	Pseudobulb (0.874 kg)	*n*-hexane fraction (SnP *n*-Hex)	5.12
			dichloromethane fraction (SnP DCM)	8.32
			ethyl acetate fraction (SnP EtOAc)	2.18
			aqueous extract (SnP Aq ext)	4.32
		Flower (0.501 kg)	*n*-hexane fraction (SnF *n*-Hex)	3.50
			dichloromethane fraction (SnF DCM)	2.10
			ethyl acetate fraction (SnF EtOAc)	3.42
			aqueous extract (SnF Aq ext)	5.12
		Leaves and Stem (2.550 kg)	*n*-hexane fraction (SnL *n*-Hex)	4.30
			dichloromethane fraction (SnL DCM)	6.02
			ethyl acetate fraction (SnL EtOAc)	2.12
			aqueous extract (SnL Aq ext)	6.30

**Table 3:** Phytochemical screening of crude fractions and extracts of three medicinal orchids of AR

S.No	Name	Alkaloids	Flavanoids	Steroids	Reducing sugars	Cardiac glycosides	Terpenoids	Anthraquinones	Tannins	Phlobatanins	Saponins
1	RrR Et_2_O	+	−	−	−	+	+	−	−	+	+
2	RrR *n* -BuOH	+	+	−	−	−	+	−	−	+	−
3	RrR Aq ext	−	−	−	−	+	−	−	−	+	+
4	RrS Et_2_O	+	+	+	+	+	+	+	−	+	+
5	RrS *n* -BuOH	+	+	+	−	−	+	−	−	+	−
6	RrS Aq ext	+	−	−	−	−	+	−	−	+	−
7	RrL Et_2_O	+	−	−	−	−	−	−	−	+	−
8	RrL *n* -BuOH	+	−	−	−	−	−	−	−	+	+
9	RrL Aq ext	+	−	−	−	−	−	−	−	+	−
10	TcR Et_2_O	+	+	+	−	−	+	+	+	+	+
11	TcR *n* -BuOH	−	−	−	+	−	+	−	−	−	−
12	TcR Aq ext	−	−	−	+	+	−	−	−	−	+
13	TcS Et_2_O	−	+	+	−	−	+	+	−	−	−
14	TcS *n* -BuOH	−	−	−	+	−	−	−	−	−	−
15	TcS Aq ext	−	−	−	+	+	−	−	−	−	−
16	TcL Et_2_O	−	+	−	−	−	−	+	+	+	−
17	TcL *n* -BuOH	−	−	−	+	−	−	−	−	−	+
18	TcL Aq ext	−	−	−	+	+	−	−	−	−	+
19	SnP *n* -Hex	+	−	−	−	−	+	−	+	−	+
20	SnP DCM	−	+	−	−	−	+	−	−	−	+
21	SnP EtOAc	+	+	−	−	−	−	+	+	−	+
22	SnP Aq ext	−	−	+	−	+	−	−	+	−	+
23	SnF *n* -Hex	+	+	−	+	+	+	−	+	+	+
24	SnF DCM	−	+	−	+	−	+	−	+	−	+
25	SnF EtOAc	+	+	+	−	+	−	+	+	−	+
26	SnF Aq ext	−	−	+	−	+	−	+	+	−	+
27	SnL *n* -Hex	+	−	−	+	−	+	−	+	−	+
28	SnL DCM	−	+	−	−	−	−	−	−	−	+
29	SnL EtOAc	+	+	−	−	+	−	−	+	−	+
30	SnL Aq ext	−	−	+	+	+	+	−	+	+	+

“+” indicates presence, “-” indicates Absence

Flavanoids, steriods, alkaloids and tannins were detected by NaOH-HCl test, Salkowski’s reaction, dragondorff reaction and ferric chloride test respectively

Additional tests were carried out to check the presence of reducing sugar, cardiac glycosides, phlobatannins, anthraqinones, saponins and terpenoids (Rajesh et al., 2010)

The antimycobacterial activity was determined by colorimetric redox indicator assay and the results were expressed in MIC values (Table 4). An analysis of the results showed that *n-*hexane fraction of the flower of *S. nepalense* (*n-*Hex SnF) exhibited most significant antimycobacterial activity against H37Rv and MDR strains with MIC of 15.7 and 42.5 μg/mL followed by Et_2_O of roots of *R. retusa* (Et_2_O RrR) with MIC of 62.5 and 125 μg/mL respectively*.* It is well documented that MIC <100 μg/mL is considered as potent while, 100–625 μg/mL represent moderate [21] antimycobacterial activity. Few more fractions including, Et_2_O fraction of RrS and TcR; EtOAc fraction of SnP and SnF; and *n*-hexane fraction of SnL exhibited moderate antimycobacterial activity (MIC 125 μg/mL) against H37Rv strain. Also, better performance demonstrated by non polar fractions might be involving the lipophilic constituents, which causes disturbance to the lipid portion of the plasma membrane, leading to a loss of permeability and leakage of intracellular materials [22]. Though, in vitro biological activities, not necessarily be transposed to clinical trials, but in vitro experiments clarify the safety aspects of a sample, to determine whether a drug candidate possesses scientific merit for further investigation.

**Table 4:** Minimum inhibitory concentration (MIC in μg/mL) of the antimycobacterial activity of crude extracts/fractions of three orchids against H37Rv and MDR strain

S.No	Extract/Fraction	MIC values against H37Rv (μg/mL)	MDR Strain-
			Tb-14,348/16 (μg/mL)
1	RrR Et_2_O	62.5	125
2	RrR *n*-BuOH	250	500
3	RrR Aq ext	>1000	>1000
4	RrS Et_2_O	125	125
5	RrS *n*-BuOH	>1000	>1000
6	RrS Aq ext	500	500
7	RrL Et_2_O	500	500
8	RrL *n*-BuOH	>1000	>1000
9	RrL Aq ext	>1000	>1000
10	TcR Et_2_O	125	104.16
11	TcR *n*-BuOH	>1000	>1000
12	TcR Aq ext	250	250
13	TcS Et_2_O	500	>1000
14	TcS *n*-BuOH	>1000	>1000
15	TcS Aq ext	1000	>1000
16	TcL Et_2_O	375	>1000
17	TcL *n*-BuOH	>1000	>1000
18	TcL Aq ext	500	>1000
19	SnP *n*-Hex	250	500
20	SnP DCM	500	500
21	SnP EtOAc	125	250
22	SnP Aq ext	>1000	>1000
23	SnF *n*-Hex	15.7	62.5
24	SnF DCM	500	>1000
25	SnF EtOAc	125	250
26	SnF Aq ext	187.5	250
27	SnL *n*-Hex	125	250
28	SnL DCM	500	500
29	SnL EtOAc	>1000	500
30	SnL Aq ext	>1000	500
	Rifampicin (positive control)	0.08	1

Experiments were carried out in triplicate and results are expressed as mean of three replicate experiments. All crude extracts or fractions were dissolved in 0.2% DMSO and distilled water. Anti-Tb drug (RIF) was prepared according to manufacturer’s instructions. Concentration range for each tested extracts or fractions were 15.6–1000 μg/mL; tested concentration range for positive control drug (RIF) was 0.25–16 μg/mL

In the second phase, the fractions were evaluated against promastigotes and intracellular amastigotes of *L. donovani* (DD8). The most efficient screening strategy for leishmanicidal activity targets i) easily cultured insect-infective promastigote stage and ii) intracellular amastigotes stage of the parasite. Chemotherapy of VL has been under-mined by drug resistance, variable efficacy, toxicity, parenteral administration, and requirement for long courses of treatment. Though, a number of plant extracts and plant derived compounds have shown promising leishmanicidal activity [23], but majority of them possesses high cytotoxicity to the normal cells. Only three fractions including, Et_2_O RrR, *n*-Hex of SnF and SnP demonstrated moderate leishmanicidal activity in the range of 50–100 μg/mL against promastigotes and 18–25 μg/mL against intracellular amastigotes (Table 5). To determine cell cytotoxicity of the active fractions, J774G8 murine macrophages were treated with different concentrations of the fractions. After 48 h, the viability was checked by MTT assay which showed negligible cytotoxic effect against macrophages at a concentration of CC_50_ values (97.2 ± 1.2, 89.4 ± 4.7 and 100.7 ± 1.7 μg/mL), which was higher than the IC_50_ values (Table 6). The cell cytotoxiciy results clearly demonstrated that the active fractions have least harmful effect on the normal cells however; the parasitic cells were affected drastically at those concentrations.

**Table 5:** In vitro leishmanicidal activity against promastigotes and intracellular amastigotes of *L. donovani*

S.No.	Extracts and fractions	IC_50_ ± SD(μg/ml) promastigotes	IC_50_ ± SD (μg/ml) amastigotes
1	RrR Et_2_O	56.04 ± 0.02	18.42 ± 0.26
2	RrR *n*-BuOH	500	125
3	RrR Aq ext	1000	1000
4	RrS Et_2_O	500	500
5	RrS *n*-BuOH	1000	500
6	RrS Aq ext	1000	500
7	RrL Et_2_O	500	250
8	RrL *n*-BuOH	1000	500
9	RrL Aq ext	300	250
10	TcR Et_2_O	250	100
11	TcR *n*-BuOH	1000	1000
12	TcR Aq ext	500	200
13	TcS Et_2_O	>1000	500
14	TcS *n*-BuOH	1000	550
15	TcS Aq ext	500	500
16	TcL Et_2_O	>1000	500
17	TcL *n*-BuOH	>1000	500
18	TcL Aq ext	>1000	>1000
19	SnP *n*-Hex	76.32 ± 2.30	23.80 ± 0.73
20	SnP DCM	1000	500
21	SnP EtOAc	500	200
22	SnP Aq ext	500	500
23	SnF *n*-Hex	65.64 ± 0.22	22.16 ± 0.99
24	SnF DCM	1000	250
25	SnF EtOAc	500	250
26	SnF Aq ext	500	500
27	SnL *n*-Hex	500	200
28	SnL DCM	1000	500
29	SnL EtOAc	>1000	500
30	SnL Aq ext	1000	1000
	Amphotericin B	0.055 ± 0.5	0.25 ± 0.48
	Miltefosine	8.11 ± 0.36	4.37 ± 0.51

The effect of different plant fractions on promastigotes (Log phase; 1 × 10^6^ cells/mL) and intracellularamastigotes were evaluated by MTT based colorimetric assay, efficacy was expressed in IC_50_ values

Standard deviation (SD) was calculated for IC_50_ < 125 μg/mL against promastigotes and amastigotes

**Table 6:** Activity against promastigotes and intracellular amastigotes of *Leishmania donovani*, cytotoxicity in J774G8 macrophage cells of active fractions

Plant fractions	Promastigote IC_50_ (μg/mL)	Amastigote IC_50_ (μg/mL)	J774.G8 CC_50_ (μg/mL)
RrR Et_2_O	56.04 ± 0.20	18.42 ± 0.26	97.2 ± 1.2
SnP *n*-Hex	76.32 ± 2.30	23.80 ± 0.73	89.4 ± 4.7
SnF *n*-Hex	65.64 ± 0.22	22.16 ± 0.99	100.7 ± 1.7

Data is presented as the mean ± SD of three independent experiments

*IC*_*50*_ inhibitory concentration of 50% parasites, *CC*_*50*_ cytotoxicity concentration of 50% cells

In commensurate with previous observations, the IC_50_ values of the active fractions were lower against amastigotes than promastigotes [24]. In this connection it is worthy to mention that screening of plants by bioassay guided fractionation only provides primary knowledge, which could be processed further by isolating compounds and evaluating their biological activity individually.

**Fig. 1:**
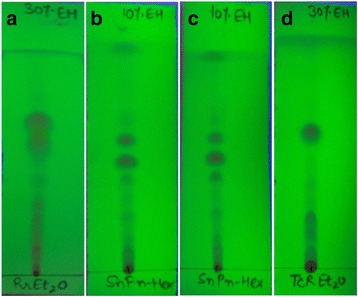
Thin Layer Chromatography (pre-coated silica TLC plates) profile of bioactive fractions: **a** diethyl ether fraction of Rr Roots; **b** *n* **-**Hex fraction of Sn Flower; **c** *n* **-**Hex fraction of Sn Pseudobulb and (**d**) diethyl ether fraction of Tc Roots The TLC plates were developed in solvent system of varying polarity, i.e. ethyl acetate: *n*-Hexane (1:9, 3:7 and 1:1), [EH]. The plates were observed under UV light (254 nm)

The present study was further extended to assess the best predictor of antibacterial activity against two gram positive and three gram negative MDR clinical isolates. The zone of inhibition values >10 mm was considered as active (Table 7). Interestingly, *n*-hexane fraction of all three parts of Sn demonstrated good antibacterial activity against *S. aureus* while, *n*-BuOH RrL and Et_2_O TcR showed significant efficiency against *Enterococcus sp.* However, most of the fractions failed to exhibit any significant activity against *Serratia sp*. Moreover, *n*-Hex SnF and Et_2_O TcR exhibited good antibacterial activity against *S. aureus* and *E. coli* with MIC values of 62.5 μg/mL and 125 μg/mL respectively while, only one fraction RrR Et_2_O showed substantial activity against *Acinetobacter sp*. with MIC of 104.16 μg/mL (Table 8). The results also revealed that aqueous extracts did not possess any antimicrobial activity against the tested strains. Similar observations were also reported from other plant species [25]. The results showed the efficacy of the plants as traditional medicine. Moreover, the active fractions were evaluated on pre-coated TLC plates with increasing concentration of ethyl acetate: *n*-Hex at 1: 9; 3: 7; and 1:1 proportions to generate the chemical profile. Maximum resolution was achieved at 3:7 proportion for Rr Et_2_O and TcR Et_2_O fractions and at 1:9 proportion for SnF *n*-Hex and SnP *n*-Hex (Fig. 1) fractions. The active fractions showed the presence of a number of distinct spots, which could be useful as a chemical profile and also in isolation of compounds.

**Table 7:** Antibacterial activity of various extracts/fractions of orchids by agar-well diffusion method

		Gram positive		Gram negative		
S. No	Extracts/fractions	*Staphylococcus aureus* (2413)	*Enterococcus sp.* (2449)	*Serratia sp.* (2442)	*Acinetobacter sp.* (2457)	*E. coli* (2461)
1	RrR Et_2_O	7.33 ± 0.57	7.66 ± 0.9	6.33 ± 0.57	13 ± 0.8	7 ± 0.81
2	RrR *n*-BuOH	6.3 ± 0.5	6.0 ± 1	6.66 ± 0.5	7.3 ± 0.5	6.66 ± 0.5
3	RrR Aq ext	6.0 ± 1	6.0 ± 1	6.0 ± 1	6.0 ± 1	6.0 ± 1
4	RrS Et_2_O	6.33 ± 0.4	7.66 ± 0.4	7.66 ± 0.4	6.66 ± 0.9	6.66 ± 0.4
5	RrS *n*-BuOH	6.66 ± 0.5	7.33 ± 0.5	6 ± 0	7.33 ± 0.5	7.0 ± 0
6	RrS Aq ext	NI	6.66 ± 0.5	6.33 ± 0.5	5.66 ± 0.5	8.0 ± 1.0
7	RrL Et_2_O	8 ± 0.8	8.33 ± 0.4	7 ± 0	8.33 ± 0.4	9.33 ± 0.4
8	RrL *n*-BuOH	6.66 ± 0.5	11.33 ± 0.5	7.33 ± 0.5	7.33 ± 0.5	8 ± 0
9	RrL Aq ext	NI	7.33 ± 0.5	6.0 ± 0	8.66 ± 0.5	7.0 ± 0
10	TcR Et_2_O	7.33 ± 0.57	12.66 ± 0.57	NI	7.66 ± 0.57	12.66 ± 0.57
11	TcR *n*-BuOH	7.66 ± 0.57	8.33 ± 0.57	NI	9.00 ± 0.00	8.33 ± 0.57
12	TcR Aq ext	8.33 ± 0.57	7.00 ± 0.00	NI	7.33 ± 0.57	7.00 ± 0.00
13	TcS Et_2_O	7.66 ± 0.57	7.66 ± 0.57	NI	7.33 ± 0.57	8.33 ± 0.57
14	TcS *n*-BuOH	7.00 ± 0.00	8.33 ± 0.57	NI	8.00 ± 0.00	8.33 ± 0.57
15	TcS Aq ext	7.33 ± 0.57	7.33 ± 0.57	NI	7.00 ± 0.00	7.33 ± 0.57
16	TcL Et_2_O	7.00 ± 0.00	7.66 ± 0.57	NI	7.00 ± 0.00	7.33 ± 0.57
17	TcL *n*-BuOH	8.33 ± 0.57	8.33 ± 0.57	NI	7.00 ± 0.00	7.00 ± 0.00
18	TcL Aq ext	7.00 ± 0.00	7.33 ± 0.57	NI	7.00 ± 0.00	7.00 ± 0.00
19	SnP *n*-Hex	12.23 ± 0.57	7.00 ± 0.00	NI	7.66 ± 0.57	9.85 ± 0.57
20	SnP DCM	7.66 ± 0.57	7.00 ± 0.00	NI	7.33 ± 0.57	8.33 ± 0.57
21	SnP EtOAc	7.00 ± 0.00	7.00 ± 0.00	NI	7.00 ± 0.00	7.33 ± 0.57
22	SnP Aq ext	7.66 ± 0.57	7.00 ± 0.00	NI	9.00 ± 0.00	8.33 ± 0.57
23	SnF *n*-Hex	12.00 ± 0.00	7.00 ± 0.00	NI	8.00 ± 0.00	11.33 ± 0.57
24	SnF DCM	8.33 ± 0.57	7.00 ± 0.00	NI	7.00 ± 0.00	7.00 ± 0.00
25	SnF EtOAc	8.33 ± 0.57	7.00 ± 0.00	NI	7.33 ± 0.57	7.00 ± 0.00
26	SnF Aq ext	7.33 ± 0.57	7.00 ± 0.00	NI	7.00 ± 0.00	7.33 ± 0.57
27	SnL *n*-Hex	11.00 ± 0.00	7.00 ± 0.00	NI	7.00 ± 0.00	9.00 ± 0.00
28	SnL DCM	9.00 ± 0.00	7.00 ± 0.00	9.00 ± 0.00	8.33 ± 0.57	7.66 ± 0.57
29	SnL EtOAc	8.00 ± 0.00	7.00 ± 0.00	8.33 ± 0.57	7.66 ± 0.57	7.33 ± 0.57
30	SnL Aq ext	7.33 ± 0.57	7.00 ± 0.00	NI	NI	NI
	Tetracycline	16.56 ± 0.58	15.71 ± 0.58	17.42 ± 0.58	15.71 ± 0.58	18.85 ± 0.58
	Gentamycin	15.76 ± 0.58	15.71 ± 0.58	11.56 ± 0.58	14.56 ± 0.58	18.79 ± 0.58

Antibacterial activity expressed as diameter of zone of inhibition in mm including 6 mm as diameter of the well

Values represented as mean ± SD of three replicates

All compounds were tested at concentration of 1 mg/mL

30 μg Tetracycline and gentamycin discs were used as positive control

NI = no inhibition

**Table 8:** Minimum Inhibitory Concentration (MIC^a^ in μg/mL) of the most potent fractions from three orchids

		Gram positive		Gram negative	
S. No	Extracts/fractions	*Staphylococcus aureus* (2413)	*Enterococcus sp.* (2449)	*Acinetobacter sp.* (2457)	*E. Coli* (2461)
1	RrR Et_2_O	250	>500	104.16	250
2	RrL *n*-BuOH	>500	250	>1000	500
3	TcR Et_2_O	500	250	500	125
4	SnP *n*-Hex	166.6	500	>1000	>1000
5	SnF *n*-Hex	125	500	500	208.33
6	SnL *n*-Hex	250	>500	>1000	>500
	Tetracyclin	0.5	16	3.7	1.9

^a^MIC determined by microdilution method and expressed in μg/mL

## Conclusion

The present study involving antimycobacterial, leishmanicidal and antibacterial activity of three orchids demonstrated *S. nepalense* as the most promising plant followed by *R. retusa* and *T. curculioides*. More specifically, the overall screening results identified *n*-Hex SnF as the most potent fraction possessing significantly high activities. Also, the fractions exhibited cell cytotoxicity well within the permissible limit. As proper phytochemical investigation of the plants has not been conducted so far, isolation and characterization of compounds could lead potential drug candidates for the experimental diseases in future.

## Abbreviations

Aq: Aqueous
AR: Arunachal Pradesh
CRI assay: Colorimetric redox indicator assay
DCM: Dichloromethane
DMSO: Dimethyl sulphoxide
Et_2_O: Diethyl ether
EtOAc: Ethyl acetate
FCS: Fetal calf serum
IC_50_: Medium inhibitory concentration
MDR: Multi-drug resistant
MIC: Minimum inhibitory concentration
MTB: *Mycobacterium tuberculosis*
MTT: 3-(4,5-dimethylthiazol-2-yl)-2,5 diphenyl tetrazolium bromide
*n*-BuOH: *n*-Butanol
*n*-Hex: *n*-Hexane
RIF: Rifampicin
Rr: *Rhynchostylis retusa*
Sn: *Satyrium nepalense*
TB: Tuberculosis
Tc: *Tropidia curculioides*
TDR: Total drug resistant
VL: visceral leishmaniasis
XDR: Extensively drug resistant
